# Supplementary material for: MicroRNA-223 Enhances Microglia-Dependent Clearance of Amyloid Beta Plaques and Ameliorates Behavioral Deficits in a Mouse Model of Alzheimer’s Disease
Source: bioRxiv. 2026 Jul 23:2026.07.20.738977. Preprint. [Version 1] doi: 10.64898/2026.07.20.738977 (PMC13419511; doi:10.64898/2026.07.20.738977)
Supplement: Supplementary file 1 [file NIHPP2026.07.20.738977v1-supplement-1.pdf]

bioRxiv preprint doi: https://doi.org/10.64898/2026.07.20.738977; this version posted July 23, 2026. The copyright holder for this preprint (which was not certified by peer review) is the author/funder, who has granted bioRxiv a license to display the preprint in perpetuity. It is made available under a CC-BY 4.0 International license.

**Figure S1: Behavioral Effects of miR-223 Modulation in Mice (Related to Figure 1)**

**A-B** Validation of miR-223 downregulation in 12-month-old $App^{NL-G-F}$ mice. **C** qPCR of cortical tissue validating miR-223 overexpression at 5 days post-treatment with a trend towards overexpression seen by day 17. **D** Open field test time in center and average velocity demonstrates no change in locomotor activity relative to no surgery (sham) animals, as well as a trend towards increased anxiety in AppNL-G-F mice treated with miR-223 inhibitor. **E-F** Barnes maze training day latency (**E**) and mistakes (**F**) in mice treated with miR-223 inhibitor. **G-H** Path length to the escape hole in mice treated with miR-223 mimic or inhibitor. **I-K** Time in target quadrant (**I**) in mice treated with miR-223 inhibitor on the 1-day probe test and latency to hole in all groups (**J**) and mistakes made (**K**) on the 1-day probe test. **L** Acquisition curves during foot shocks during day 1 of the contextual fear conditioning (CFC) paradigm (left = miR-223 mimic, right = miR-223 inhibitor). **M** miR-223 inhibitor does not change freezing behavior during the tone test relative to scrambled control. **N** miR-223 overall does not change freezing during the context text relative to scrambled control.

**Figure S2: Role of miR-223 on Amyloidosis in $App^{NL-G-F}$ Mice (Related to Figure 1)**

**A** Representative AmyloGlo staining images of experimental groups in the cortex and hippocampus. **B-C** Quantification of the number of Aβ plaques in the cortex and hippocampus shows significant reduction in mimic treated mice, with no change in inhibitor treated mice. **D** Representative ThioS staining images of experimental groups in the cortex and hippocampus. **E-F** ThioS+ staining in the cortex and hippocampus shows no change with miR-223 inhibitor treatment.

bioRxiv preprint doi: https://doi.org/10.64898/2026.07.20.738977; this version posted July 23, 2026. The copyright holder for this preprint (which was not certified by peer review) is the author/funder, who has granted bioRxiv a license to display the preprint in perpetuity. It is made available under a CC-BY 4.0 International license.

**Figure S3: Transcriptomic Characterization of miR-223 (Related to Figure 1)**

**A** GSEA enrichment of miR-223 validated target genes changed by miR-223 at day 5 in the cortex and hippocampus. **B** GSEA enrichment of miR-223 validated target genes changed by miR-223 treatment at day 17 in the cortex and hippocampus. **C-D** Number of up and downregulated DEGs in the noted treatment groups. **E** Volcano plot of DEGs between miR-223 versus scrambled control treated $App^{NL\text{-}G\text{-}F}$ mice at 17 days post-treatment in the cortex. **F** Pathway enrichment in the cortex of day 17 miR-223 treated mice reveals downregulation of immune response. **G** Heatmap of miR-223 protected genes in the cortex of day 17 miR-223 treated mice. **H** Volcano plot of DEGs between miR-223 versus scrambled control treated $App^{NL\text{-}G\text{-}F}$ mice at 17 days post-treatment in the hippocampus. **I** Pathway enrichment in the hippocampus of day 17 miR-223 treated mice reveals upregulation for synapse organization. **J** Heatmap of miR-223 protected genes in the hippocampus of day 17 miR-223 treated mice. **K** Correlation of Log2FoldChange values between miR-223 vs scrambled control in $App^{NL\text{-}G\text{-}F}$ mice and $App^{NL\text{-}G\text{-}F}$ vs WT mice in the cortex and hippocampus. The top left quadrant (red) represents genes downregulated in $App^{NL\text{-}G\text{-}F}$ vs WT mice and upregulated in miR-223 vs scrambled control in $App^{NL\text{-}G\text{-}F}$ mice. The bottom left quadrant (blue) represents genes upregulated in $App^{NL\text{-}G\text{-}F}$ vs WT mice and downregulated in miR-223 vs scrambled control in $App^{NL\text{-}G\text{-}F}$ mice.

bioRxiv preprint doi: https://doi.org/10.64898/2026.07.20.738977; this version posted July 23, 2026. The copyright holder for this preprint (which was not certified by peer review) is the author/funder, who has granted bioRxiv a license to display the preprint in perpetuity. It is made available under a CC-BY 4.0 International license.

**Figure S4: Multiplex Immunohistochemical Staining of miR-223 Treated Mice (Related to Figure 2)**

**A** Representative whole hemi-brain images of multiplex IHC-staining in wild type scrambled control (WT), App$^{NL\text{-}G\text{-}F}$ scrambled control and App$^{NL\text{-}G\text{-}F}$ miR-223 treated mice. **B-C** Distribution of peri-plaque SYP+ area between scrambled control and miR-223 treated mice (n = 30 plaques from n = 6 mice) in the cortex and hippocampus. Each dot represents one plaque. **D-E** Distribution of peri-plaque LAMP1+ area between scrambled control and miR-223 treated mice (n = 30 plaques from n = 6 mice) in the cortex and hippocampus. Each dot represents one plaque.

**Figure S5: Characterization of Microgliosis in miR-223 Treated Mice (Related to Figure 3)**

**A** Assessment of microglial ramification via Sholl analysis reveals increased complexity in the cortex of miR-223 treated mice. **B** Treatment time course for assessing microglial changes in miR-223 treated mice. **C** Representative IBA1 staining of day 17 post-treatment WT scrambled control, App$^{NL\text{-}G\text{-}F}$ scrambled control and App$^{NL\text{-}G\text{-}F}$ miR-223. **D-E** Ramification index is decreased between WT and App$^{NL\text{-}G\text{-}F}$ mice and is not changed by miR-223 treatment at day 17 post treatment. **F** Representative images and quantification of peri-plaque IBA1+ area in the cortex and hippocampus at day 17 demonstrates increased clustering around plaques in the cortex. **G** Western blot quantification of microglia receptors involved in Aβ phagocytosis in the cortex and hippocampus of day 17 post-treatment mice, including AXL, MER, CD11c, and TREM2. **H-I** Western blot quantification of NLRP3 and ASC in the cortex and hippocampus of day 5 treated mice. **J** Representative immunofluorescence staining demonstrating restricted expression of CD11c surrounding amyloid plaques. **K** ThioS quantification of amyloid plaques in the cortex and hippocampus shows PLX5622 treatment itself does not alter Aβ pathology after 12 days of treatment.

bioRxiv preprint doi: https://doi.org/10.64898/2026.07.20.738977; this version posted July 23, 2026. The copyright holder for this preprint (which was not certified by peer review) is the author/funder, who has granted bioRxiv a license to display the preprint in perpetuity. It is made available under a CC-BY 4.0 International license.

**Figure S6: Characterization of miR-223 in iPSC-Derived Microglia (Related to Figure 4)**

**A** Experimental design for primary microglia studies. **B** Validation of miR-223 overexpression in mimic treated primary microglia via qPCR. **C** Gene expression changes in primary microglia are associated with inflammation and microglial function. **D** Normalized phagocytic activity measured via Vybrant assay in primary microglia treated with miR-223 or scrambled control. **E** Validation of miR-223 overexpression in mimic treated iMGs prior to RNA-seq. **F** Volcano plot of significant DEGs changed with miR-223 overexpression in iMGs. **G** Heatmap of microglial and immune genes downregulated by miR-223 treatment measured via RNA-seq. **H** Validation of miR-223 knockdown in inhibitor treated iMGs prior to RNA-seq. **I** Volcano plot of significant DEGs changed with miR-223 knockdown in iMGs. **J** Heatmap of microglial and immune genes upregulated by miR-223 knockdown measured via RNA-seq. **K** Gene expression for microglial genes *IL6, IL1B, NLRP3*, and *ITGAX* with miR-223 inhibitor and Aβo. **L** Pathway enrichment of direct targets downregulated by miR-223 mimic treatment reveals enrichment of endosome-associated genes. **M** Heatmap of top direct targets downregulated by miR-223 mimic treatment. **N** Pathway enrichment of direct targets upregulated by miR-223 inhibitor treatment reveals enrichment of immune, cytokine, and endosome-associated genes. **O** Heatmap of top direct targets upregulated by miR-223 inhibitor treatment.

bioRxiv preprint doi: https://doi.org/10.64898/2026.07.20.738977; this version posted July 23, 2026. The copyright holder for this preprint (which was not certified by peer review) is the author/funder, who has granted bioRxiv a license to display the preprint in perpetuity. It is made available under a CC-BY 4.0 International license.

**Figure S7: Characterization of miR-223 in iPSC-derived Neurons**

**A** Validation of miR-223 overexpression in mimic-treated iNeurons by qPCR. **B** Volcano plot of DEGs changed with miR-223 mimic treatment. **C** Pathway enrichment of top upregulated and downregulated neuronal DEGs upon treatment by miR-223 mimic. **D** Heatmap of synaptic signaling genes and their change with miR-223 treatment. **E** Validation of miR-223 knock-down in inhibitor treated iNeurons by qPCR. **F** Volcano plot of DEGs changed with miR-223 mimic treatment. **G** Pathway enrichment of top upregulated and downregulated neuronal DEGs upon treatment by miR-223 inhibitor. **H** Heatmap of synaptic signaling genes and their change with miR-223 treatment. **I** Heatmap of top miR-223 target genes that are downregulated by mimic treatment in iNeurons. **J** Heatmap of top miR-223 target genes that are upregulated by inhibitor treatment in iNeurons. **K** Pathway enrichment of significantly downregulated target genes with mimic treatment. **L** Pathway enrichment of significantly upregulated target genes with inhibitor treatment. **M** Overlap between direct targets decreased with mimic treatment and upregulated with inhibitor treatment. **N** Fold change RNA-seq values of 7 overlapping genes identified in **M**. **O** Peak calcium flux miR-223 mimic and inhibitor treated iNeurons upon glutamate stimulation.

**Figure S8: Validation and Behavioral Testing of AAV Mice**

**A** qPCR validation of miR-223 levels in AAV mice. **B** Assessment of overlap between tdTomato and glial stains. White arrows represent tdTomato+ microglia. **C** Time in center and average velocity in the open field tests associated with AAV treatment groups. **D-E** Barne's maze latency to escape hole over trial (**D**) and probe (**E**) days. **F-G** Contextual fear conditioning test across learning, tone and context tests. **I-J** Representative images and quantification of the effect of miR-223 on glial and synaptic markers in WT mice. **K** Microglia numbers per 10 $mm^2$ FOV.

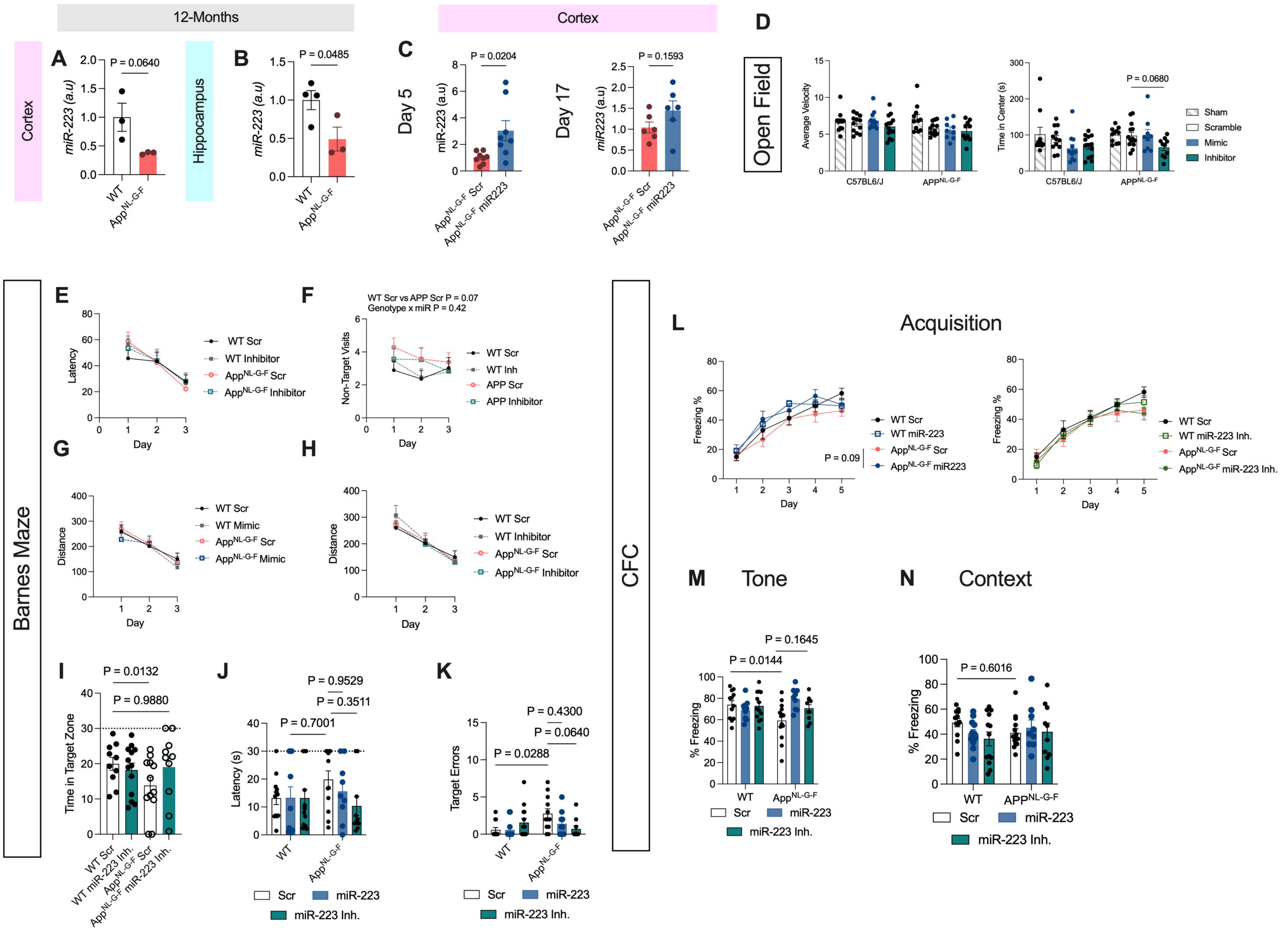

12-Months
Cortex
Hippocampus
A
B
C
D
miR-223 (a.u)
P = 0.0640
P = 0.0485
WT
App^NL-G-F
Day 5
Day 17
P = 0.0204
P = 0.1593
App^NL-G-F Scr
App^NL-G-F miR223
Open Field
Average Velocity
Time in Center (s)
P = 0.0680
C57BL6/J
APP^NL-G-F
Sham
Scramble
Mimic
Inhibitor
Barnes Maze
E
F
G
H
Latency
Day
WT Scr
WT Inhibitor
App^NL-G-F Scr
App^NL-G-F Inhibitor
WT Scr vs APP Scr P = 0.07
Genotype x miR P = 0.42
Non-Target Visits
WT Inh
APP Scr
APP Inhibitor
Distance
WT Mimic
App^NL-G-F Mimic
I
J
K
Time in Target Zone
P = 0.0132
P = 0.9880
WT Scr
WT miR-223 Inh.
App^NL-G-F Scr
App^NL-G-F miR-223 Inh.
Latency (s)
P = 0.7001
P = 0.9529
P = 0.3511
Target Errors
P = 0.0288
P = 0.4300
P = 0.0640
Scr
miR-223
miR-223 Inh.
CFC
L
Acquisition
Freezing %
P = 0.09
WT miR-223
App^NL-G-F miR223
WT miR-223 Inh.
App^NL-G-F miR-223 Inh.
M
Tone
N
Context
% Freezing
P = 0.0144
P = 0.1645
P = 0.6016

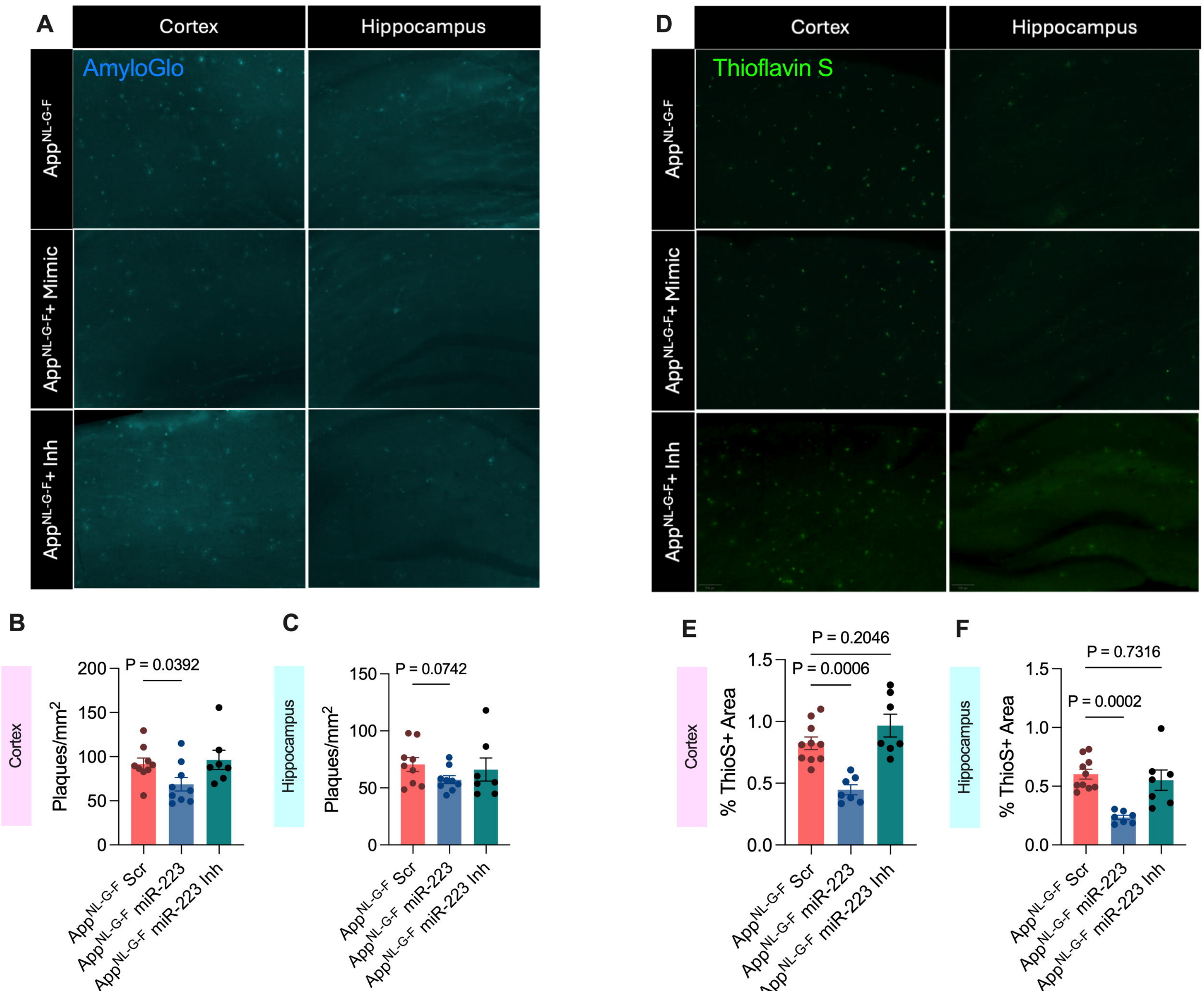

A
Cortex
Hippocampus
AmyloGlo
App$^{NL-G-F}$
App$^{NL-G-F}$+ Mimic
App$^{NL-G-F}$+ Inh
D
Cortex
Hippocampus
Thioflavin S
App$^{NL-G-F}$
App$^{NL-G-F}$+ Mimic
App$^{NL-G-F}$+ Inh
B
Cortex
Plaques/mm$^2$
P = 0.0392
0
50
100
150
200
App$^{NL-G-F}$ Scr
App$^{NL-G-F}$ miR-223
App$^{NL-G-F}$ miR-223 Inh
C
Hippocampus
Plaques/mm$^2$
P = 0.0742
0
50
100
150
App$^{NL-G-F}$ Scr
App$^{NL-G-F}$ miR-223
App$^{NL-G-F}$ miR-223 Inh
E
Cortex
% ThioS+ Area
P = 0.2046
P = 0.0006
0.0
0.5
1.0
1.5
App$^{NL-G-F}$ Scr
App$^{NL-G-F}$ miR-223
App$^{NL-G-F}$ miR-223 Inh
F
Hippocampus
% ThioS+ Area
P = 0.7316
P = 0.0002
0.0
0.5
1.0
1.5
App$^{NL-G-F}$ Scr
App$^{NL-G-F}$ miR-223
App$^{NL-G-F}$ miR-223 Inh

(which was not certified by peer review) is the author/funder, who has granted bioRxiv a license to display the preprint in p
available under a CC-BY 4.0 International license.

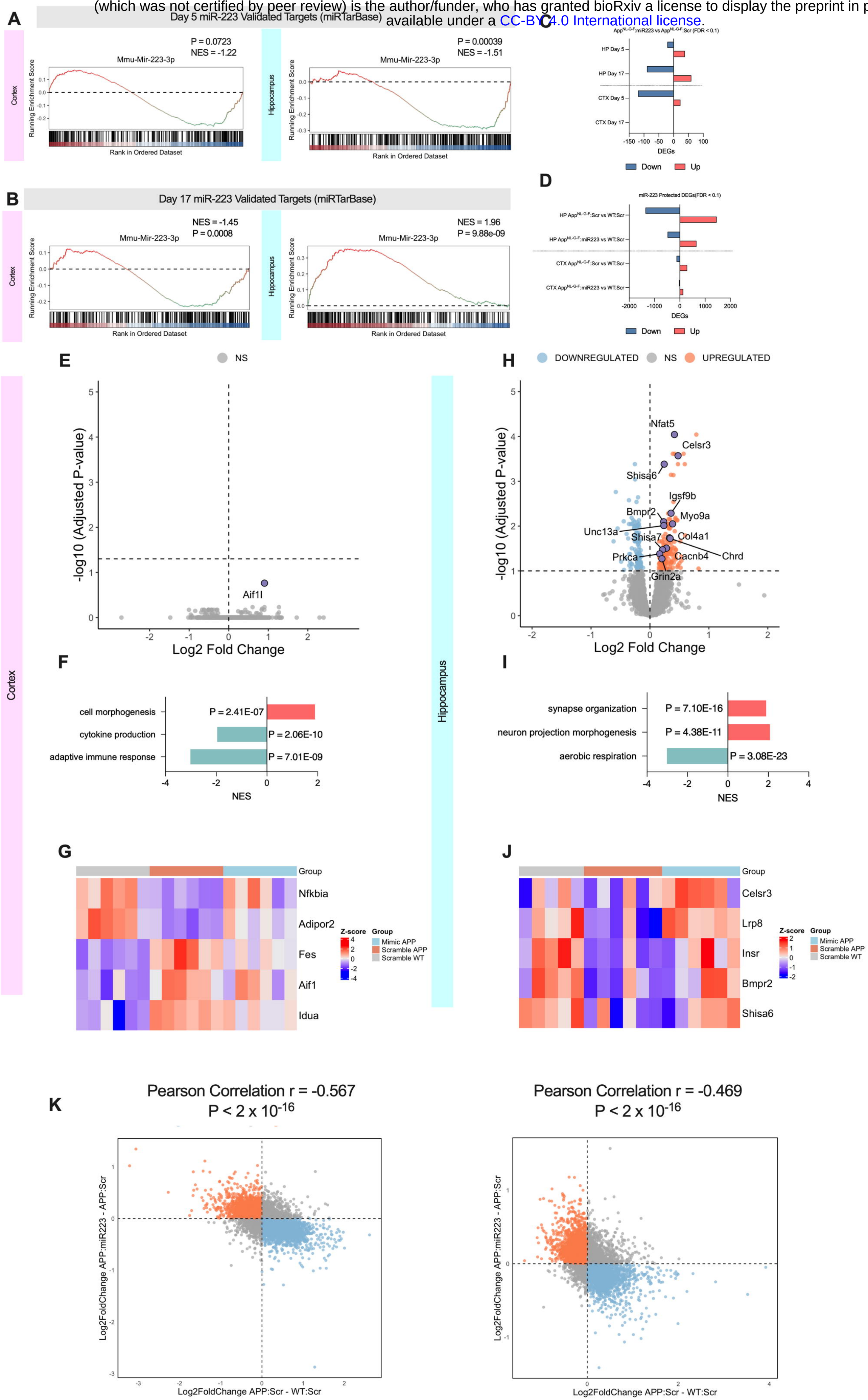

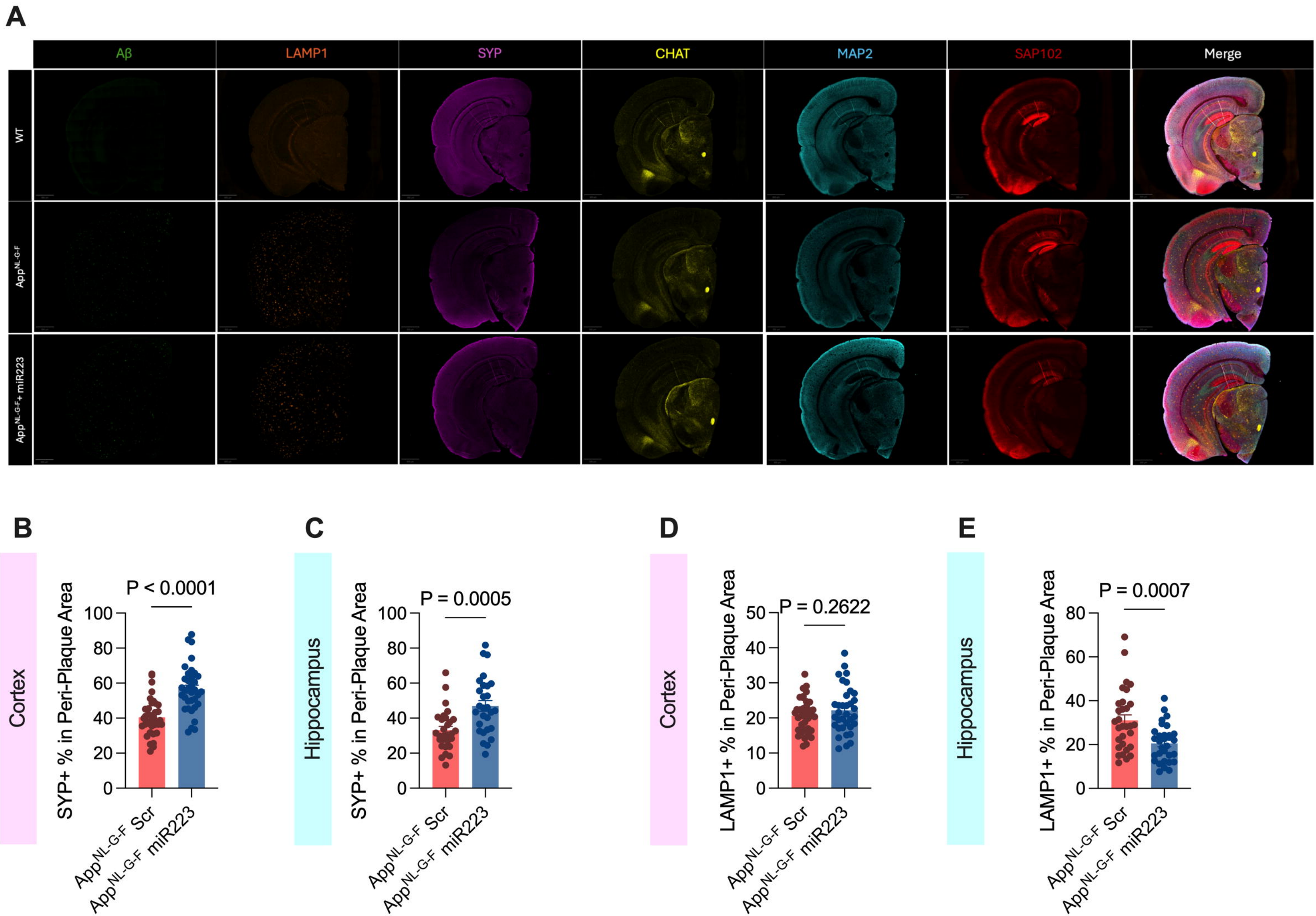
A
Aβ
LAMP1
SYP
CHAT
MAP2
SAP102
Merge
WT
App$^{NL-G-F}$
App$^{NL-G-F}$+ miR223
B
Cortex
SYP+ % in Peri-Plaque Area
P < 0.0001
App$^{NL-G-F}$ Scr
App$^{NL-G-F}$ miR223
C
Hippocampus
SYP+ % in Peri-Plaque Area
P = 0.0005
App$^{NL-G-F}$ Scr
App$^{NL-G-F}$ miR223
D
Cortex
LAMP1+ % in Peri-Plaque Area
P = 0.2622
App$^{NL-G-F}$ Scr
App$^{NL-G-F}$ miR223
E
Hippocampus
LAMP1+ % in Peri-Plaque Area
P = 0.0007
App$^{NL-G-F}$ Scr
App$^{NL-G-F}$ miR223

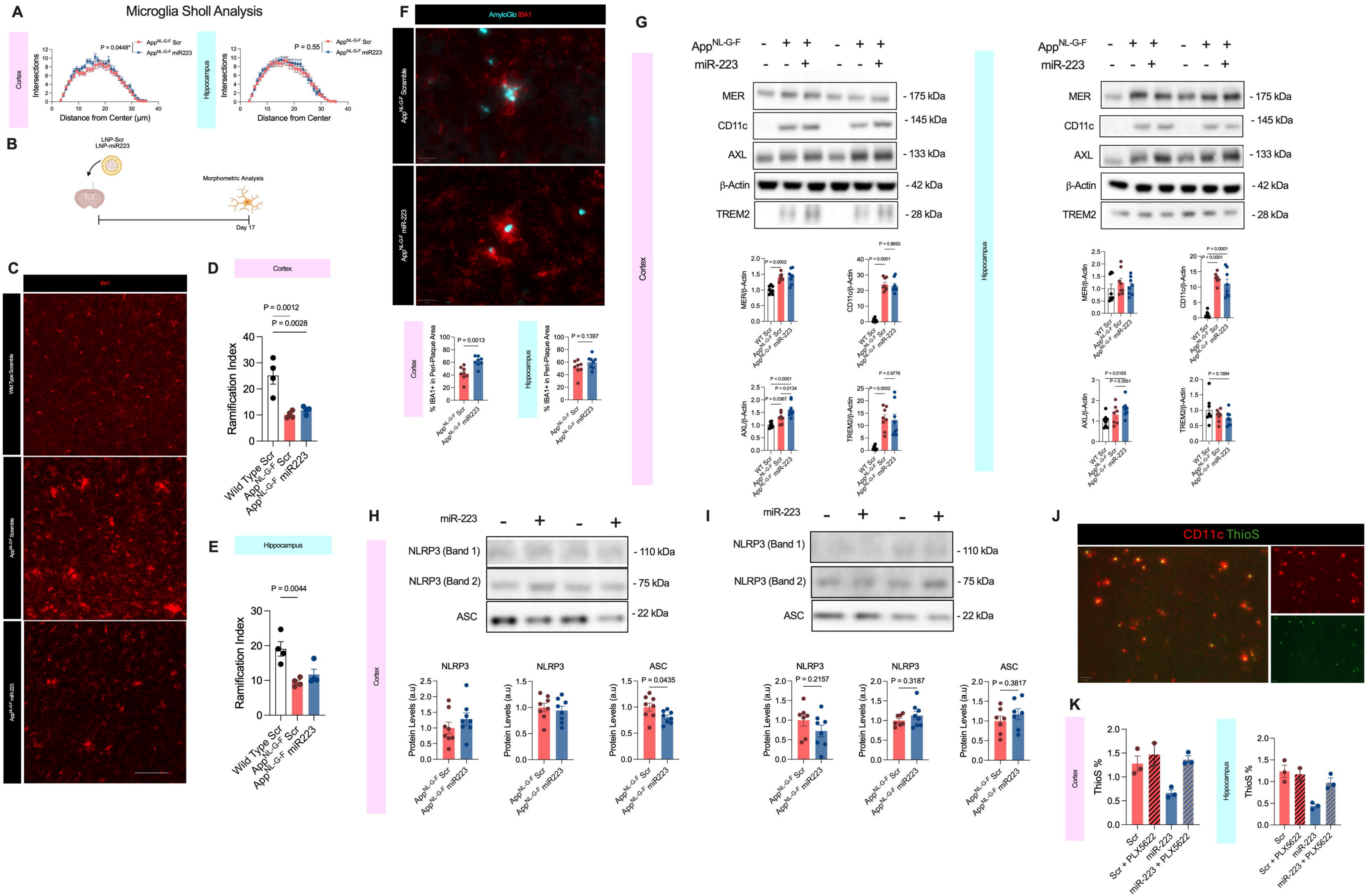
A
Microglia Sholl Analysis
Cortex
Hippocampus
P = 0.0448*
P = 0.55
Intersections
Distance from Center (μm)
Distance from Center
B
LNP-Scr
LNP-miR223
Morphometric Analysis
Day 17
C
IBA1
Wild Type Scramble
D
Cortex
P = 0.0012
P = 0.0028
Ramification Index
E
Hippocampus
P = 0.0044
Ramification Index
F
AmyloGlo IBA1
% IBA1+ in Peri-Plaque Area
P = 0.0013
P = 0.1397
G
miR-223
MER
CD11c
AXL
β-Actin
TREM2
- 175 kDa
- 145 kDa
- 133 kDa
- 42 kDa
- 28 kDa
MER/β-Actin
CD11c/β-Actin
AXL/β-Actin
TREM2/β-Actin
H
miR-223
NLRP3 (Band 1)
NLRP3 (Band 2)
ASC
- 110 kDa
- 75 kDa
- 22 kDa
NLRP3
ASC
Protein Levels (a.u)
P = 0.0435
I
P = 0.2157
P = 0.3187
P = 0.3817
J
CD11c ThioS
K
ThioS %
Scr
Scr + PLX5622
miR-223
miR-223 + PLX5622

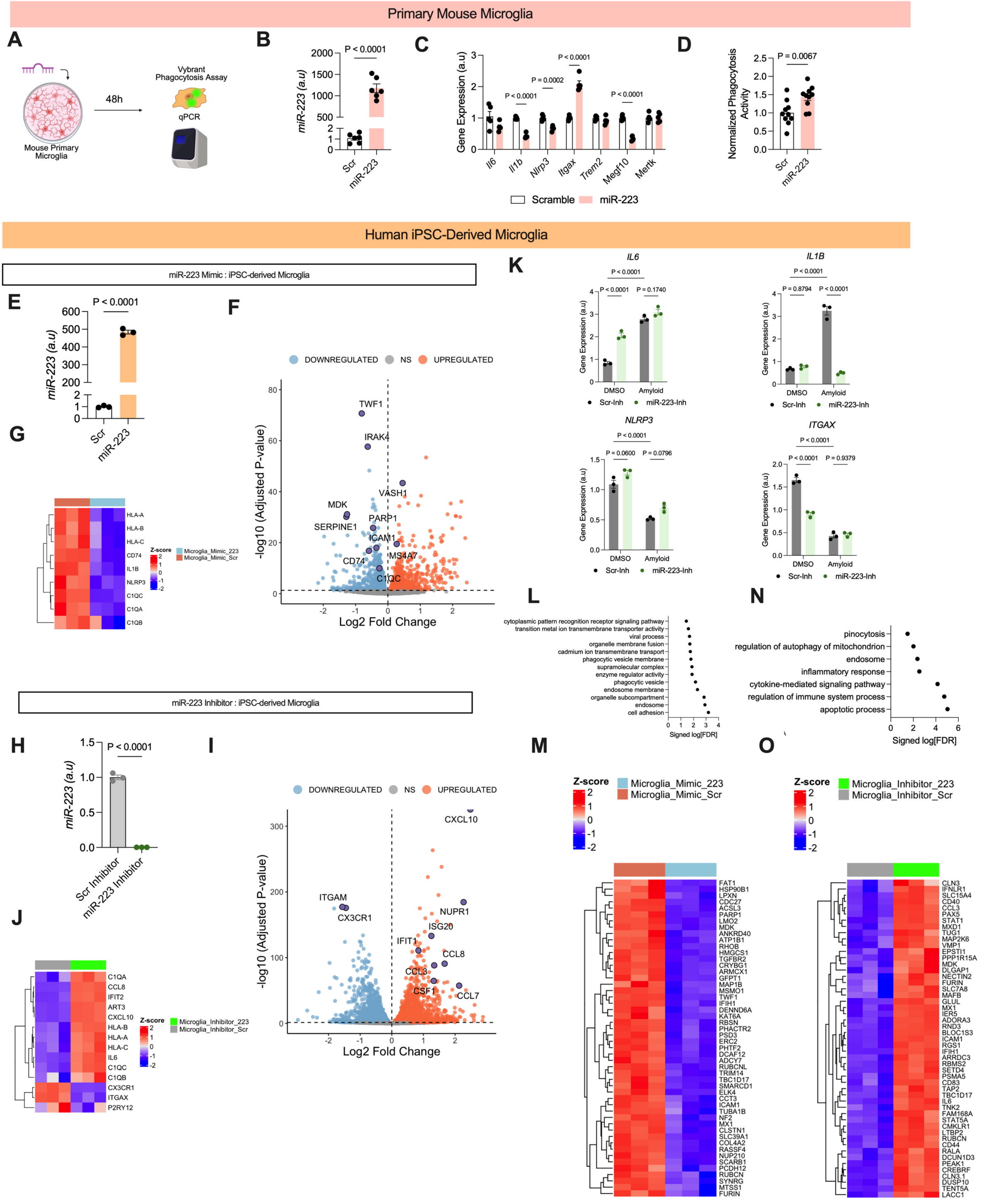
Primary Mouse Microglia
A
Vybrant Phagocytosis Assay
48h
qPCR
Mouse Primary Microglia
B
P < 0.0001
miR-223 (a.u)
Scr
miR-223
C
Gene Expression (a.u)
P < 0.0001
P = 0.0002
P < 0.0001
P < 0.0001
P < 0.0001
Il6
Il1b
Nlrp3
Itgax
Trem2
Megf10
Mertk
Scramble
miR-223
D
P = 0.0067
Normalized Phagocytosis Activity
Scr
miR-223
Human iPSC-Derived Microglia
miR-223 Mimic : iPSC-derived Microglia
E
P < 0.0001
miR-223 (a.u)
Scr
miR-223
F
DOWNREGULATED
NS
UPREGULATED
-log10 (Adjusted P-value)
Log2 Fold Change
TWF1
IRAK4
VASH1
MDK
PARP1
SERPINE1
ICAM1
MS4A7
CD74
C1QC
G
HLA-A
HLA-B
HLA-C
CD74
IL1B
NLRP3
C1QC
C1QA
C1QB
Z-score
Microglia_Mimic_223
Microglia_Mimic_Scr
K
IL6
P < 0.0001
P < 0.0001
P = 0.1740
Gene Expression (a.u)
DMSO
Amyloid
Scr-Inh
miR-223-Inh
IL1B
P < 0.0001
P = 0.8794
P < 0.0001
NLRP3
P < 0.0001
P = 0.0600
P = 0.0796
ITGAX
P < 0.0001
P < 0.0001
P = 0.9379
L
cytoplasmic pattern recognition receptor signaling pathway
transition metal ion transmembrane transporter activity
viral process
organelle membrane fusion
cadmium ion transmembrane transport
phagocytic vesicle membrane
supramolecular complex
enzyme regulator activity
phagocytic vesicle
endosome membrane
organelle subcompartment
endosome
cell adhesion
Signed log[FDR]
N
pinocytosis
regulation of autophagy of mitochondrion
endosome
inflammatory response
cytokine-mediated signaling pathway
regulation of immune system process
apoptotic process
Signed log[FDR]
miR-223 Inhibitor : iPSC-derived Microglia
H
P < 0.0001
miR-223 (a.u)
Scr Inhibitor
miR-223 Inhibitor
I
DOWNREGULATED
NS
UPREGULATED
-log10 (Adjusted P-value)
Log2 Fold Change
CXCL10
ITGAM
CX3CR1
NUPR1
ISG20
IFIT1
CCL8
CCL3
CSF1
CCL7
J
C1QA
CCL8
IFIT2
ART3
CXCL10
HLA-B
HLA-A
HLA-C
IL6
C1QC
C1QB
CX3CR1
ITGAX
P2RY12
Z-score
Microglia_Inhibitor_223
Microglia_Inhibitor_Scr
M
Z-score
Microglia_Mimic_223
Microglia_Mimic_Scr
FAT1
HSP90B1
LPXN
CDC27
ACSL3
PARP1
LMO2
MDK
ANKRD40
ATP1B1
RHOB
HMGCS1
TGFBR2
CRYBG1
ARMCX1
GFPT1
MAP1B
MSMO1
TWF1
IFIH1
DENND6A
KAT6A
RBSN
PHACTR2
PSD3
ERC2
PHTF2
DCAF12
ADCY7
RUBCNL
TRIM14
TBC1D17
SMARCD1
ELK4
CCT3
ICAM1
TUBA1B
NF2
MX1
CLSTN1
SLC39A1
COL4A2
RASSF4
NUP210
SCARB1
PCDH12
RUBCN
SYNRG
MTSS1
FURIN
O
Z-score
Microglia_Inhibitor_223
Microglia_Inhibitor_Scr
CLN3
IFNLR1
SLC15A4
CD40
CCL3
PAX5
STAT1
MXD1
TUG1
MAP2K6
VMP1
EPSTI1
PPP1R15A
MDK
DLGAP1
NECTIN2
FURIN
SLC7A8
MAFB
GLUL
MX1
IER5
ADORA3
RND3
BLOC1S3
ICAM1
RGS1
IFIH1
ARRDC3
RBMS2
SETD4
PSMA5
CD83
TAP2
TBC1D17
IL6
TNK2
FAM168A
STAT5A
CMKLR1
LTBP2
RUBCN
CD44
RALA
DCUN1D3
PEAK1
CREBRF
CLN3.1
DUSP10
TENT5A
LACC1

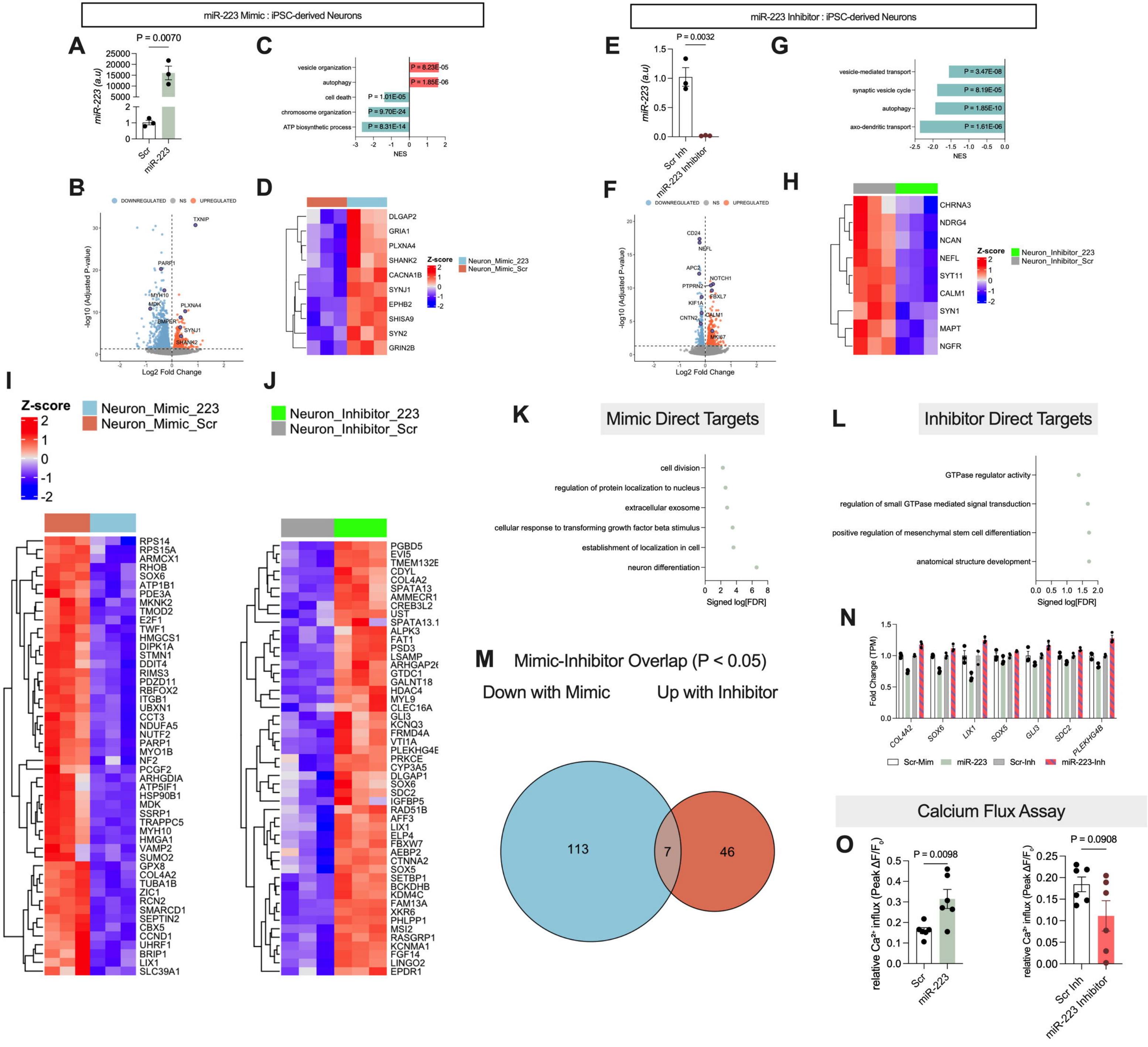
miR-223 Mimic : iPSC-derived Neurons
miR-223 Inhibitor : iPSC-derived Neurons
A
P = 0.0070
miR-223 (a.u)
Scr
miR-223
B
DOWNREGULATED
NS
UPREGULATED
TXNIP
PARP1
MYH10
MDK
BMPER
PLXNA4
SYNJ1
SHANK2
-log10 (Adjusted P-value)
Log2 Fold Change
C
vesicle organization
autophagy
cell death
chromosome organization
ATP biosynthetic process
P = 8.23E-05
P = 1.85E-06
P = 1.01E-05
P = 9.70E-24
P = 8.31E-14
NES
D
DLGAP2
GRIA1
PLXNA4
SHANK2
CACNA1B
SYNJ1
EPHB2
SHISA9
SYN2
GRIN2B
Z-score
Neuron_Mimic_223
Neuron_Mimic_Scr
E
P = 0.0032
miR-223 (a.u)
Scr Inh
miR-223 Inhibitor
F
DOWNREGULATED
NS
UPREGULATED
CD24
NEFL
APC2
NOTCH1
PTPRN2
FBXL7
KIF1A
CALM1
CNTN2
MKI67
-log10 (Adjusted P-value)
Log2 Fold Change
G
vesicle-mediated transport
synaptic vesicle cycle
autophagy
axo-dendritic transport
P = 3.47E-08
P = 8.19E-05
P = 1.85E-10
P = 1.61E-06
NES
H
CHRNA3
NDRG4
NCAN
NEFL
SYT11
CALM1
SYN1
MAPT
NGFR
Z-score
Neuron_Inhibitor_223
Neuron_Inhibitor_Scr
I
Z-score
Neuron_Mimic_223
Neuron_Mimic_Scr
RPS14
RPS15A
ARMCX1
RHOB
SOX6
ATP1B1
PDE3A
MKNK2
TMOD2
E2F1
TWF1
HMGCS1
DIPK1A
STMN1
DDIT4
RIMS3
PDZD11
RBFOX2
ITGB1
UBXN1
CCT3
NDUFA5
NUTF2
PARP1
MYO1B
NF2
PCGF2
ARHGDIA
ATP5IF1
HSP90B1
MDK
SSRP1
TRAPPC5
MYH10
HMGA1
VAMP2
SUMO2
GPX8
COL4A2
TUBA1B
ZIC1
RCN2
SMARCD1
SEPTIN2
CBX5
CCND1
UHRF1
BRIP1
LIX1
SLC39A1
J
Neuron_Inhibitor_223
Neuron_Inhibitor_Scr
PGBD5
EVI5
TMEM132E
CDYL
COL4A2
SPATA13
AMMECR1
CREB3L2
UST
SPATA13.1
ALPK3
FAT1
PSD3
LSAMP
ARHGAP26
GTDC1
GALNT18
HDAC4
MYL9
CLEC16A
GLI3
KCNQ3
FRMD4A
VTI1A
PLEKHG4B
PRKCE
CYP3A5
DLGAP1
SOX6
SDC2
IGFBP5
RAD51B
AFF3
LIX1
ELP4
FBXW7
AEBP2
CTNNA2
SOX5
SETBP1
BCKDHB
KDM4C
FAM13A
XKR6
PHLPP1
MSI2
RASGRP1
KCNMA1
FGF14
LINGO2
EPDR1
K
Mimic Direct Targets
cell division
regulation of protein localization to nucleus
extracellular exosome
cellular response to transforming growth factor beta stimulus
establishment of localization in cell
neuron differentiation
Signed log[FDR]
L
Inhibitor Direct Targets
GTPase regulator activity
regulation of small GTPase mediated signal transduction
positive regulation of mesenchymal stem cell differentiation
anatomical structure development
Signed log[FDR]
M
Mimic-Inhibitor Overlap (P < 0.05)
Down with Mimic
Up with Inhibitor
113
7
46
N
Fold Change (TPM)
COL4A2
SOX6
LIX1
SOX5
GLI3
SDC2
PLEKHG4B
Scr-Mim
miR-223
Scr-Inh
miR-223-Inh
Calcium Flux Assay
O
P = 0.0098
P = 0.0908
relative Ca²⁺ influx (Peak ΔF/F₀)
Scr
miR-223
Scr Inh
miR-223 Inhibitor

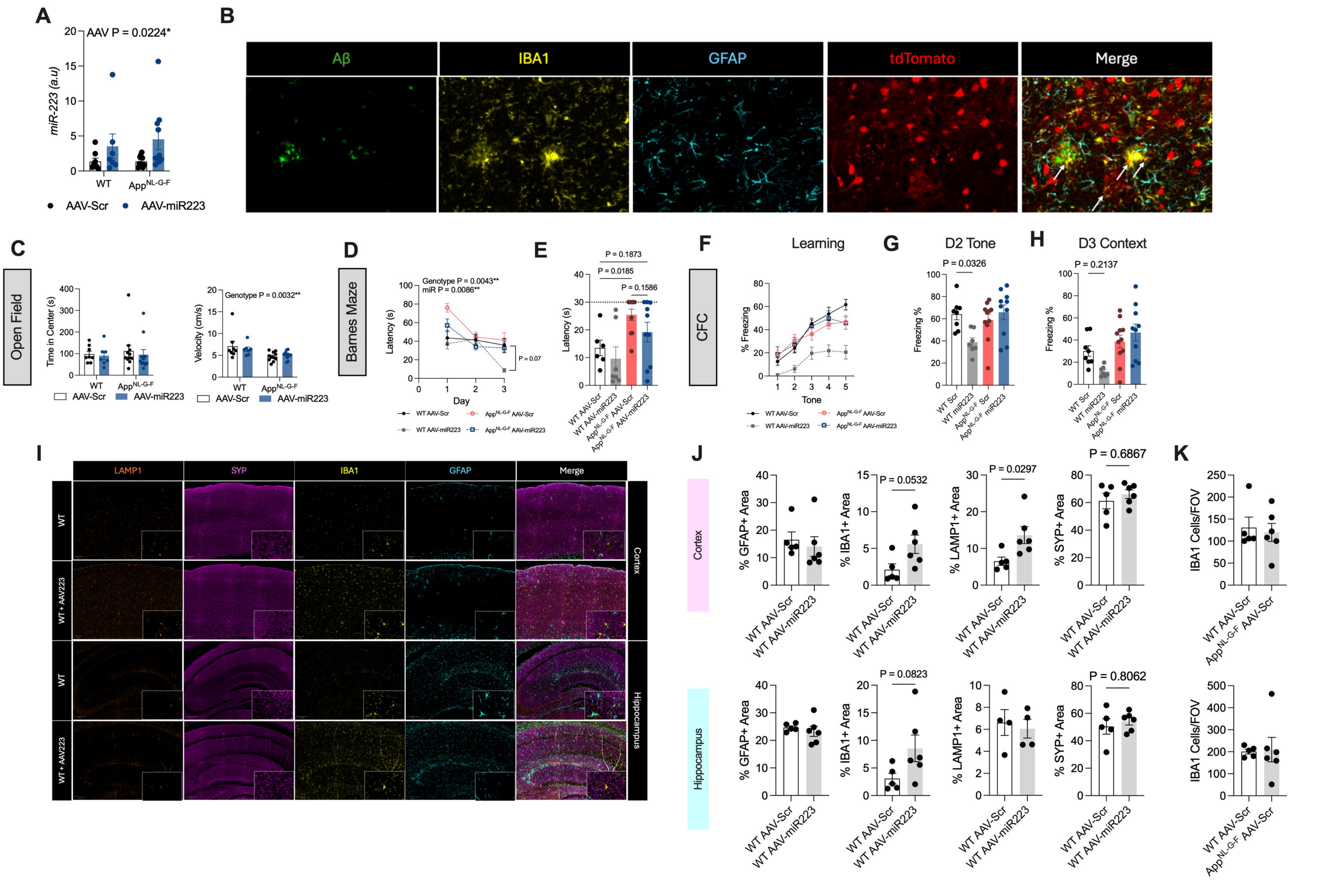
A
AAV P = 0.0224*
miR-223 (a.u)
WT
App NL-G-F
AAV-Scr
AAV-miR223
B
Aβ
IBA1
GFAP
tdTomato
Merge
C
Open Field
Time in Center (s)
Velocity (cm/s)
Genotype P = 0.0032**
D
Barnes Maze
Genotype P = 0.0043**
miR P = 0.0086**
Latency (s)
Day
P = 0.07
WT AAV-Scr
WT AAV-miR223
App NL-G-F AAV-Scr
App NL-G-F AAV-miR223
E
P = 0.1873
P = 0.0185
P = 0.1586
F
CFC
Learning
% Freezing
Tone
G
D2 Tone
P = 0.0326
Freezing %
H
D3 Context
P = 0.2137
I
LAMP1
SYP
IBA1
GFAP
Merge
WT
WT + AAV223
Cortex
Hippocampus
J
Cortex
Hippocampus
% GFAP+ Area
% IBA1+ Area
% LAMP1+ Area
% SYP+ Area
P = 0.0532
P = 0.0297
P = 0.6867
P = 0.0823
P = 0.8062
WT AAV-Scr
WT AAV-miR223
K
IBA1 Cells/FOV
App NL-G-F AAV-Scr
